# The Emotional Lexicon of Individuals Diagnosed with Antisocial Personality Disorder

**DOI:** 10.1007/s10936-012-9237-z

**Published:** 2013-01-22

**Authors:** Barbara Gawda

**Affiliations:** Department of Psychology, Maria Curie-Sklodowska University, Plac Litewski 5, 20-080 Lublin, Poland

**Keywords:** Antisocial personality disorder, Anxiety, Hate, Emotional lexicon, Love

## Abstract

This study investigated the specific emotional lexicons in narratives created by persons diagnosed with antisocial personality disorder (ASPD) to test the hypothesis that individuals with ASPD exhibit deficiencies in emotional language. Study participants consisted of 60 prison inmates with ASPD, 40 prison inmates without ASPD, and 60 men without antisocial tendencies who described situations involving love, hate and anxiety depicted by photographs. The lexical choices made in the narratives were analyzed, and a comparison of the three groups revealed differences between the emotional narratives of inmates with ASPD, inmates without ASPD, and the control group. Although the narratives of the individuals with ASPD included more words describing emotions and higher levels of emotional intensity, the valence of these words was inappropriate. The linguistic characteristics of these narratives were associated with high levels of psychopathy and low emotional reactivity.

## Introduction

In linguistics, the words and expressions of a language form its lexicon or vocabulary, with the emotional lexicon consists of the words or expressions used to describe emotions. This study investigated the emotional lexicon of individuals diagnosed with antisocial personality disorder (ASPD) to explore and understand their problems with affect. Previous research has produced conflicting findings. Although some studies have found that the emotional language of individuals with antisocial personality disorder is rich and expressive, others have found that it is impoverished (Brinkley et al. 1999; Eichler 1965; Hare et al. 1988; Keltikangas-Jarvinene 1982).

One long-standing research concern has been the extent to which antisocial personality disorder is related to antisocial behavior, psychopathy and criminal behavior. The literature has found that these syndromes are correlated but not equivalent (Patrick 2007). However, many theories regarding the genesis, mechanisms, and characteristics of the psychopathic or antisocial personality have claimed that the affective deficits are common in individuals diagnosed with these disorders (Hare 1998, 2003). The literature typically reports that psychopaths exhibit emotional impairments, such as dysfunctional affective processing (a lack of insight into emotions and inability to analyze past experience), weak emotional control, predominantly negative affect, and the inability to experience more complex emotions—in particular, closeness, respect, trust, and guilt. Affective functioning in the antisocial personality is regulated by a multilevel system characterized by ruthlessness, narcissism, hostility, manipulation, and sensation-seeking (Hiatt and Newman 2007; Lykken 2007; Pauthus and Williams 2002). Researchers have acknowledged that the general characterization of affective disorders in psychopaths as a lack of complex emotions or lack of anxiety might be misleading regarding individuals who exhibit specific disorders. For example, the data concerning lack of anxiety among psychopaths is inconsistent, and the most recent findings have indicated that these individuals do not exhibit a lack of anxiety (Lynam and Derefinko 2007).

Research on the emotional language of psychopaths might better explain affective disorders in these individuals (Gawda 2010; Rieber and Vetter 1994). Because mental representations of the world and other people are expressed through linguistic concepts, linguistic analysis is valuable for understanding human affective processing (Alford 2005; Sternberg 2005; Sternberg 2006), and emotional discourse provides considerable information regarding an individual’s experience, emotions, sensations, and perception of the world and others (van Dijk 1997).

Recent research on the language used by psychopaths has found that individuals with psychopathic personalities create less structured narratives that lack temporal perspective (Keltikangas-Jarvinene 1982) and do not describe the emotional context or focus on negative aspects of the situation (Dolan and Anderson 2002). Psychopaths exhibit difficulties in storing and recalling emotional information (Dolan and Fullam 2005), and they exhibit better memory for information with negative affect than for information with positive affect (Christianson et al. 1996; Christianson and Loftus 1991).

Analysis of the emotional content of psychopaths’ texts indicated that they are less cohesive (Eichler 1965), although a lack of text coherence was not a consistent finding and Brinkley et al. (1999) concluded that only offenders with high anxiety levels produced less coherent texts. Texts produced by individuals with ASPD include many negative statements, are self-focused, and include emotionally intense vocabulary (Eichler 1965; Gawda 2010). It has been hypothesized that the number of negative statements is linked to emotional schemata and scripts (Gawda 2010) and that psychopaths use the language of action without reference to the emotional meaning of the words (Hare 1998; Hare et al. 1988). Research on psychopaths’ language (Hare et al. 1988; Hiatt et al. 2002; Williamson et al. 1991) found that psychopaths display difficulty with semantic interpretation as well as in differentiating emotional information from neutral information. This disorder produces problems in interpreting the meaning of the situation (Brinkley et al. 2005; Lorenz and Newman 2002).

Because the process of constructing emotional knowledge is closely related to its expression through language, linguistic analysis might identify primary emotional characteristics. The present study focused on the emotions of love, hate and anxiety because previous studies have found that these feelings are problematic in individuals with antisocial personality disorder and that an inability to feel love or intimacy based on love is a characteristic of these individuals (Blair et al. 1997). These individuals are also unable to feel anxiety but exhibit the capacity to experience emotions connected with hate, such as anger or hostility (Brinkley et al. 2005; Hare 1996, 1998).

To assess the emotional lexicon, the present study investigated monologic narrative discourse to determine whether there were differences in the emotional lexicons of love, hate and anxiety between prison inmates diagnosed with ASPD, inmates without ASPD, and a control group. We expected that inmates with antisocial personality disorder would employ a more restricted affective lexicon that displayed higher levels of intensity and negative valence. We also hypothesized that the emotional lexicons of individuals with antisocial personality disorder would be related to affective and personality traits such as trait anxiety, psychopathy, and emotional reactivity.

## Method

### Participants

Participants included three groups of individuals. The first group (ASPD) consisted of 60 prison inmates with a diagnosis of antisocial personality disorder who did not exhibit other mental and organic disorders; the diagnosis was based on individuals’ records using the DSM-IV-TR criteria. Individuals in this group had been convicted of multiple serious crimes against health, life and public order. The second group (nonASPD) consisted of 40 prison inmates without a diagnosis of antisocial personality disorder or neuropsychological and psychiatric impairments, who had been convicted of the same types of multiple crimes and were serving similar sentences; the diagnosis was based on interviews and individuals’ records. Both groups of inmates were similar in age. The control group (CONT) consisted of 60 adult male students with low scores on the Pd scale of the MMPI who did not display antisocial tendencies based on the DSM-IV-TR (see Table 1).

**Table 1:** Descriptive statistics and comparisons between three groups: ASPD ($N = 60$), nonASPD ($N = 40$), and CONT ($N = 60$)

Variables	ASPD $M$ (*SD*)	nonASPD $M$ (*SD*)	CONT $M$ (*SD*)	$F_{(2,157)}$
Age	35.5 (11)$^\mathrm{a}$	34.2 (10)$^\mathrm{a}$	33.5 (1.45)$^\mathrm{a}$	1.44 n.s.
Education	10.27 (1.80)$^\mathrm{a}$	10.40 (1.50)$^\mathrm{a}$	10.44 (1.50)$^\mathrm{a}$	.66 n.s.
Full scale IQ	102 (10)$^\mathrm{a}$	99 (9)$^\mathrm{a}$	100 (9)$^\mathrm{a}$	1.04 n.s.
Verbal IQ	99 (6)$^\mathrm{a}$	97 (7)$^\mathrm{a}$	99 (8)$^\mathrm{a}$	1.81 n.s.
Performance IQ	103 (6)$^\mathrm{a}$	101 (8)$^\mathrm{a}$	101 (8)$^\mathrm{a}$	1.23 n.s.
Working memory	52 (9)$^\mathrm{a}$	53.5 (9)$^\mathrm{a}$	53 (8.5)$^\mathrm{a}$	1.78 n.s.
Verbal comprehension	53.5 (8)$^\mathrm{a}$	54 (7)$^\mathrm{a}$	56 (7)$^\mathrm{a}$	1.04 n.s.
STAI trait	44.5 (8.84)$^\mathrm{a}$	41.5 (8.40)$^\mathrm{b}$	38.26 (6.82)$^\mathrm{c}$	12.36***
FCB-TI (ER)	8.73 (4.22)$^\mathrm{a}$	11.43 (4.09)$^\mathrm{b}$	7.97 (4.12)$^\mathrm{a}$	9.83***
MMPI-Pd	87.1 (10.4)$^\mathrm{a}$	59.6 (12.5)$^\mathrm{b}$	46.9 (10.3)$^\mathrm{c}$	9.98***
MMPI-Ma	75.4 (9.6)$^\mathrm{a}$	57.2 (8.91)$^\mathrm{b}$	48.2 (9.2)$^\mathrm{c}$	9.12***

$^\mathrm{abc }$ Same superscript for means in a row indicate no significant difference by Tukey’s *post hoc* test.

$$^*p<.05,\,^{**} p<.01,\,^{***} p \quad <.001$$

### Procedure

The initial phase of the investigation was performed in the prisons, and the second in the schools. After prisoner records were analyzed, interviews were conducted and background data were collected. Five measures were obtained. Four measures were based on widely used psychological instruments: the WAIS-R, the MMPI, the STAI (Wrześniewski et al. 2002) and the Temperament Inventory (FCB-TI; Strelau and Zawadzki 1997). The fifth measure was obtained by asking each participant to create three narratives about love, hate and anxiety based on three photographs.

### Measures

Two MMPI scales—the Psychopathy (Pd) and Hypomania (Ma) scales—were used because antisocial individuals have higher scores on these two scales.

The Wechsler Adult Intelligence Scale-Revised (WAIS-R) is a general test of intelligence with verbal and performance components. There are 11 subtests, and the verbal component consists of six verbal subtests. The Verbal and Performance scores were employed in the analysis, and the Working Memory Index and Verbal Comprehension Index were also taken into consideration.

The Polish version of the State Trait Anxiety Inventory (STAI; Wrześniewski et al. 2002) was used in this study, but only the Trait Anxiety score was included in the analysis.

The Emotional Reactivity scale of the Temperament Inventory (FCB-TI; Strelau and Zawadzki 1997), which measures the tendency to react strongly to emotional stimuli, was used in this study. Cronbach’s alpha coefficients ranged from 77 to 84, confirming the validity and reliability of the instrument (Strelau and Zawadzki 1997).

Based on a pilot study, three photographs (one associated with love, one with hate, and one with anxiety) were used. Participants in the pilot study rated the extent to which 24 photographs (8 for each emotion) expressed love, hate, or anxiety. The photographs that exhibited the highest significant levels of inter-rater agreement were selected for the study (Kendall’s W = 0.93 for love; Kendall’s W = 0.86 for hate; and Kendall’s W = 0.88 for anxiety). In the present study, every participant was shown each of the three photographs associated with love, hate or anxiety and asked to write a story based on the photograph. Each photograph was shown separately, and the participant was given the following instructions: “Look at this picture. Imagine that you are in the picture, how you would feel, and then write a story about the photograph”. The time allowed for writing was not limited, but controlled. The participants produced 480 stories (160 describing love, 160 describing hate, and 160 describing anxiety). Three psychologists analyzed the narratives. The number of lexical indicators in a story was determined, and scores were then averaged for each participant. The following emotional lexicon measures were used: the total number of the emotional words and expressions (e.g., *happiness, joy, I love you*); the negative or positive valence of the emotional words or expressions, which was represented by the number of the negative emotion words (e.g., *disappointed, unhappy*) and the number of the positive emotion words (e.g., *contented, good*); the number of emotional words or expressions at each level of intensity, which was graded as weak (e.g., *calm*), medium (e.g., *happy*), or high (e.g., *very *or *extremely happy*); and the number of different types of emotional words (i.e., nouns, verbs, or adjectives). These measures were obtained for all narratives.

## Results

Participants’ intelligence test scores were used to control for the influence of intellectual level on participants’ narratives. The three groups did not exhibit significant differences in verbal intelligence (see Table 1). A one-way analysis of variance with the factors of age, education, and intelligence found no statistically significant differences between the three groups in age, years of education, IQ, Verbal IQ Working Memory Index, and Verbal Comprehension (see Table 1). The differences in the groups’ narratives did not appear to be due to the verbal or linguistic capabilities of the participants but were based on emotional and personality characteristics. Kruskal-Wallis tests were performed to assess differences between the three groups (and Mann-Whitney test to assess differences between each two groups) in the lexical features of participants’ narratives about love, hate and anxiety (see Tables 2,  3, and  4).

**Table 2:** Differences in the lexical features of love narratives for the ASPD ($N = 60$), nonASPD ($N = 40$), and CONT ($N = 60$) groups

Lexical features	ASPD $M$ (*SD*)	nonASPD $M$ (*SD*)	CONT $M$ (*SD*)	$H_{(2)}$
Emotional words	6.88 (4.03)$^\mathrm{a}$	3.97 (3.14)$^\mathrm{b}$	5.25 (3.31)$^\mathrm{c}$	6.65**
Positive words	5.86 (0.69)$^\mathrm{a}$	2.50 (1.40)$^\mathrm{b}$	4.93 (1.16)$^\mathrm{a}$	6.65**
Negative words	1.02 (1.03)$^\mathrm{a}$	.47 (.41)$^\mathrm{b}$	.37 (.21)$^\mathrm{b}$	16.86***
Intensity: weak	1.68 (.72)$^\mathrm{a}$	1.50 (.70)$^\mathrm{a}$	1.98 (.49)$^\mathrm{a}$	1.81 n.s.
Intensity: medium	1.88 (.98)$^\mathrm{a}$	1.21 (.91)$^\mathrm{b}$	1.87 (.90)$^\mathrm{a}$	1.23 n.s.
Intensity: high	3.26 (.79)$^\mathrm{a}$	1.26 (.89)$^\mathrm{b}$	1.40 (.71)$^\mathrm{b}$	13.50***
Nouns	1.78 (.91)$^\mathrm{a}$	1.10 (.91)$^\mathrm{a}$	1.48 (.47)$^\mathrm{a}$	1.59 n.s.
Verbs	2.13 (.82)$^\mathrm{a}$	1.20 (.90)$^\mathrm{b}$	1.47 (.78)$^\mathrm{b}$	3.70*
Adjectives	2.47 (.80)$^\mathrm{a}$	1.28 (.27)$^\mathrm{b}$	1.86 (.73)$^\mathrm{c}$	7.23***

$^\mathrm{abc }$ Same superscript for means in a row indicate no significant difference by Mann-Whitney test.

$$^{*}p<.05,\,^{**} p<.01,^{***} p <.001$$

**Table 3:** Differences in the lexical features of hate narratives for the ASPD ($N = 60$), nonASPD ($N = 40$), and CONT ($N = 60$) groups

Lexical features	ASPD $M$ (*SD*)	nonASPD $M$ (*SD*)	CONT $M$ (*SD*)	$H_{(2)}$
Emotional words	6.14 (4.39)$^\mathrm{a}$	4.15 (2.50)$^\mathrm{b}$	4.91 (2.52)$^\mathrm{b}$	8.83***
Positive words	4.61 (1.93)$^\mathrm{a}$	1.09 (1.40)$^\mathrm{b}$	.93 (1.16)$^\mathrm{b}$	8.65***
Negative words	1.53 (1.40)$^\mathrm{a}$	3.06 (1.41)$^\mathrm{b}$	3.98 (2.21)$^\mathrm{b}$	9.35***
Intensity: weak	2.68 (.72)$^\mathrm{a}$	1.50 (.70)$^\mathrm{b}$	1.98 (.49)$^\mathrm{b}$	3.01*
Intensity: medium	1.88 (.98)$^\mathrm{a}$	1.28 (.91)$^\mathrm{a}$	1.87 (.90)$^\mathrm{a}$	1.29 n.s.
Intensity: high	1.58 (1.79)$^\mathrm{a}$	1.36 (.89)$^\mathrm{a}$	1.40 (.71)$^\mathrm{a}$	1.98 n.s.
Nouns	2.39 (2.13)$^\mathrm{a}$	1.23 (1.54)$^\mathrm{b}$	1.58 (1.47)$^\mathrm{b}$	5.82**
Verbs	2.34 (1.62)$^\mathrm{a}$	2.02 (1.62)$^\mathrm{a}$	1.90 (1.11)$^\mathrm{a}$	.75 n.s.
Adjectives	1.00 (.80)$^\mathrm{a}$	.79 (.79)$^\mathrm{a}$	1.13 (1.25)$^\mathrm{a}$	1.95 n.s.

$^\mathrm{abc }$ Same superscript for means in a row indicate no significant difference by Mann-Whitney test.

$$^{*}p<.05,\,^{**} p<.01,\,^{***} p<.001$$

**Table 4:** Differences in the lexical features of anxiety narratives for the ASPD ($N = 60$), nonASPD ($N = 40$), and CONT ($N = 60$) groups

Lexical features	ASPD *M (SD)*	nonASPD *M (SD)*	CONT *M (SD)*	$F_{(2)}$
Emotional words	5.35 (2.38)$^\mathrm{a}$	3.58 (1.14)$^\mathrm{b}$	4.51 (2.31)$^\mathrm{c}$	4.34*
Positive words	.98 (.69)$^\mathrm{a}$	.13 (.40)$^\mathrm{b}$	.30 (.16)$^\mathrm{b}$	9.97***
Negative words	4.37 (2.41)$^\mathrm{a}$	3.25 (1.41)$^\mathrm{b}$	4.38 (2.21)$^\mathrm{a}$	4.60*
Intensity: weak	1.80 (.98)$^\mathrm{a}$	1.90 (.87)$^\mathrm{a}$	2.11 (.98)$^\mathrm{a}$	1.12 n.s.
Intensity: medium	1.90 (.89)$^\mathrm{a}$	.90 (.56)$^\mathrm{a}$	1.28 (.68)$^\mathrm{a}$	2.03 n.s.
Intensity: high	1.65 (.78)$^\mathrm{a}$	.78 (.59)$^\mathrm{b}$	1.12 (.65)$^\mathrm{b}$	4.90*
Nouns	1.55 (1.12)$^\mathrm{a}$	.94 (.92)$^\mathrm{a}$	1.26 (1.32)$^\mathrm{a}$	1.49 n.s.
Verbs	2.01 (1.75)$^\mathrm{a}$	1.10 (.98)$^\mathrm{b}$	1.50 (1.34)$^\mathrm{b}$	6.31**
Adjectives	1.44 (1.03)$^\mathrm{a}$	1.17 (1.01)$^\mathrm{a}$	1.30 (.92)$^\mathrm{a}$	.45 n.s.

$^\mathrm{abc}$ Same superscript for means in a row indicate no significant difference by Mann-Whitney test.

$$^{*}p<.05,\,^{**} p<.01,\,^{***} p <.001$$

Comparisons of the lexical elements in the three types of narratives created by the ASPD group, the nonASPD group, and the control group indicated that the groups were significantly different from each other.

The ASPD group used more emotional words and emotional expressions in their narratives than the nonASPD group and the control group. In the narratives describing love, the ASPD group used more positive and negative emotional words than the other two groups.

Their narratives describing love also contained more verbs (e.g., *wait, meet, be, hug, kiss, take*) and adjectives (e.g., *fantastic, beautiful, wonderful, happy, content, joyful*). The ASPD group described love more expressively than the other groups. They achieved this effect by using significantly more adjectives than the other two groups and by using certain verbs that might underlie the actions. The narratives describing hate written by the ASPD group contained more positive than negative emotional words. Compared to the other groups, their stories about hate included more nouns (e.g., *anger, quarrel, rage, hostility, claims, calmness, agreement, understanding*), and the emotional expressions used were less intense. For the narratives describing hate, the three groups did not differ in the use of verbs, adjectives, or emotional expressions of medium, and high intensity, although the nonASPD group and controls described hate more negatively. That means that they recognized the appropriate valence of hate.

For the narratives describing anxiety, the ASPD group used more positive and negative emotion words. Their emotional descriptions of anxiety were more intense than those made by the other groups. They used more verbs in the stories about anxiety (e.g., *feel, be, miss, wait, can, come, long for, dream*).

Multiple regression analyses assessed the contributions of psychopathy, hypomania, emotional reactivity and trait anxiety to the emotional lexicons of the narratives that described love, hate, and anxiety. For narratives describing love, psychopathy explained 13 % of the variance in the use of emotional words ($\beta =.28, p<.01), 19\,\%$ of the variance in positive and negative emotional words ($\beta =.32, p<.01),$ and 9 % of the variance in the use of verbs and adjectives ($\beta =.27, p<.01)$ for verbs, $\beta =.36, p<.01$ for adjectives; $(F_{(4,155)} = 4.23, p<.01).$ Trait anxiety explained 10 % of the variance in the intensity of expression for narratives describing love ($\beta =.32,p<.01)$ and 8 % of the variance in the use of verbs ($\beta =.25, p<.01; F_{(4,155)} = 4.45, p<.01).$ Emotional reactivity and hypomania did not significantly contribute to the model for narratives describing love.

Psychopathy explained 20 % of the variance in the use of positive words for the narratives describing hate ($\beta =.53, p<.001).$ Emotional reactivity explained 14 % of the variance in the frequency of positive emotional words ($\beta = -.40, p<.001)$ and 14 % of the variance in the frequency of nouns ($\beta = -.29, p<.01; F_{(4,155)} = 4.65, p<.01).$ Trait anxiety explained 7 % of the variance in the frequency of nouns ($\beta =.24, p<.01; F_{(4,155)} = 4.65, p<.01).$

A multiple regression analysis found that psychopathy, emotional reactivity and trait anxiety were significantly associated with the lexical features of the narratives describing anxiety. Psychopathy and emotional reactivity explained 23 % of the variance in the frequency of emotional words in narratives describing anxiety ($\beta _\mathrm{Pd }=.24, p<.01; \beta _\mathrm{ER }= -.22, p<.01; F_{(4,155)} = 4.25, p<.01).$ Psychopathy and emotional reactivity explained 25 % of the variance in the frequency of negative emotional words ($\beta _\mathrm{Pd}=.49, p<.001; \beta _\mathrm{ER}= -.16, p<.01; F_{(4,155)} = 8.93, p<.001).$ Psychopathy and trait anxiety explained 20 % of the variance in the frequency of positive emotional words in the narratives about anxiety ($\beta _\mathrm{Pd}=.28, p<.01; \beta _\mathrm{TA }=.29, p<.01; F_{(4,155)} = 7.33, p<.001).$ Psychopathy explained 12 % of the variance in the frequency of verbs ($\beta =.33, p<.01, F_{(4,155)} = 4.13, p<.01).$ Hypomania did not contribute significantly to the models for either the hate or anxiety narratives.

## Discussion

The study findings confirmed that individuals with ASPD used ambivalent emotional expressions in narrative descriptions of love, hate, and anxiety. Narratives describing love expressed both positive and negative emotions (e.g., happiness, joy, sadness, anger and aversion). Narratives describing hate also exhibited ambivalence in emotional valence, with the actors experiencing diverse emotions such as anger, hostility and aversion, as well as joy and calmness. The characteristics of actors in the narratives describing hate produced by individuals with ASPD were highly positive. The narratives describing hate produced by individuals with ASPD included many verbs that inappropriately expressed actions. The vocabulary used to describe anxiety was also ambivalent, with individuals simultaneously expressing joy, fear, loneliness, and guilt. The study results are consistent with the previous findings indicating a lack of coherence of the texts produced by psychopathic individuals (Brinkley et al. 1999; Eichler 1965). The ambivalence of the emotional lexicon revealed the ambivalent affective experiences of individuals with ASPD. These results indicated that the emotional experience of individuals with ASPD was ambiguous and vague because they were unable to precisely identify the valence of the relevant emotions, understand the basis of the emotional situation, or react in an emotionally appropriate way. They employed many strategies that distorted the meaning of complex emotions. Although they possessed an emotional vocabulary, their lexicons were composed of vague, ambivalent and ambiguous concepts. The ambivalence regarding emotions affected their ability to appropriately recognize the goals, needs, intentions, and causes of different emotional situations. These affective impairments cause problems in interpreting the meaning of the emotional situation (these problems are reported by many researchers, for example Brinkley et al. 2005; Hiatt et al. 2002; Lorenz and Newman 2002; Williamson et al. 1991).

In part, the study results support the view that individuals with ASPD experience negative feelings more often than positive feelings (Dolan and Fullam 2005; Patrick 2007). Recent studies have found that individuals with ASPD are able to feel anger and rage but unable to experience fear and dissatisfaction (Blair et al. 1997; Blair et al. 2002; Blair et al. 2006; Kosson 2002) and recognize the facial expression of anger more quickly and accurately than the expression of other emotions. In the present study, anger was described in all of the emotional narratives, including the narratives describing love and anxiety. The results of the present study, which found that individuals with ASPD experienced emotions ambivalently, are consistent with research that has investigated the behavioral approach and behavioral inhibition systems that provide the physiological mechanisms underlying ambivalence in individuals with psychopathic personality (Levenson et al. 1995; Newman et al. 2005). These studies have found that individuals with ASPD exhibit higher levels of approach activation, and lower levels of approach inhibition (Newman et al. 2005; Sutker and Allain 2001), as well as stronger reactions to neutral compared to emotional stimuli. Although they are able to experience the emotions, they quickly activate control mechanisms that introduce ambiguity in regard to the valence of emotions. Individuals with ASPD have been found to produce ambiguous responses to suboptimal stimuli (Bradley et al. 1996; Levenson et al. 2000; Müller et al. 2003).

Hate is an extremely negative feeling (Berkowitz 2005), and an individual who feels hate negatively evaluates the hated object (Alford 2005). The findings of the present study revealed that people with ASPD did not perceive hate as hypothesized. On the contrary, the participants without ASPD and from the control group perceived hate adequately, as negative emotion (Sternberg 2005). In the narratives describing hate, they described themselves as good, conciliatory, and able to solve problems. Their emotional expressions were intense and included many positive comments regarding their behavior in the situation. This finding may illustrate ASPD personality traits, such as charm and narcissism (Hare and Neumann 2007). The positive view of the self that was presented indicated the use of defense mechanisms such as negation, underestimation, belittlement of the others and overvaluation of themselves, which were expressed by descriptions of their own competence and ability to solve problems. The composition of the narratives describing hate exhibited affective dysfunctions in the individuals with ASPD, such as the lack of emotional insight and the inability to analyze past experience. The dysfunctional insight has extremely negative and serious consequences (Hare and Neumann 2007).

Anxiety has a negative valence, and involves fear, hopelessness, incapacity, indecisiveness, sadness, loneliness, and general nervousness. The ASPD group’s descriptions of anxiety revealed that they were unable to unambiguously express the emotions related to anxiety. They expressed ambivalent emotions and employed different defense mechanisms such as idealization, negation, and dogmatic opinions, which were reflected in the language they used to elaborate on the affective information. Their perception of the world was inflexible (Luchins and Luchins 1982) and exhibited the cognitive rigidity that is typical in individuals with antisocial personality disorder. The emotional language of individuals in the ASPD group revealed cognitive distortions and impairment in the elaboration of information. They tended to overestimate their abilities, underestimate the abilities of other people, and engage in sweeping generalizations (e.g., *I am able to solve every type of problem*). Individuals with ASPD employed specific strategies, such as selecting information that overestimated their own skills and ignoring unfavorable aspects of the situation. They possessed a rich emotional vocabulary and affective lexicon, which allowed them to describe emotions (Gawda 2010). However, although their affective lexicon was rich, their narrative forms were well structured, and they possessed emotional imagination, they found it difficult to clearly recognize the affective valence of their emotions. This might lead to problems, such as the inability to appropriately evaluate an emotional event, the inability to understand the meaning of an emotion, and the inability to predict one’s own behavior. When an emotion is ambiguous, it is difficult for the individual to control behaviors related to the emotion (Hiatt and Newman 2007).

Noteworthy, that psychopathy interacts with the other factors as emotional reactivity and trait anxiety. These two variables and psychopathic traits were significantly associated with the emotional lexicon. We might suppose that a specific constellation of psychopathy, emotional reactivity and trait anxiety determines the emotional lexicon. Which is why, for example the individuals without ASPD with high emotional reactivity, and medium anxiety, might produce the specific syntactic constructions, with for example less verbs in their expressions of anxiety, or less adjectives in their love stories (Gawda 2010; Izard et al. 1999-2000; Ragatt 2006).

Further studies on the emotional language in psychopaths and ASPD individuals might be focused on a use of the combined methods, both qualitative and the objective measures such as RT response. It might give an better insight into the cognitive and affective processes.
